# Photo-based External Quality Assessment of Malaria rapid diagnostic tests in a non-endemic setting

**DOI:** 10.1371/journal.pone.0201622

**Published:** 2018-08-31

**Authors:** Kris Vernelen, Barbara Barbé, Philippe Gillet, Marjan Van Esbroeck, Bernard China, Jan Jacobs

**Affiliations:** 1 Quality of Medical Laboratories, Institute of Public Health, Sciensano, Brussels, Belgium; 2 Institute of Tropical Medicine, Antwerp, Belgium; 3 KU Leuven, Department of Microbiology and Immunology, Leuven, Belgium; Instituto Rene Rachou, BRAZIL

## Abstract

**Introduction:**

In non-endemic settings, expertise in malaria microscopy is limited and rapid diagnostic tests (RDTs) are an adjunct to malaria diagnosis.

**Aim:**

We performed an External Quality Assessment (EQA) on reading and interpretation of malaria RDTs in a non-endemic setting.

**Methods:**

Participants were medical laboratories in Belgium and the Grand Duchy of Luxembourg using malaria RDTs; they received (i) 10 high-resolution photographs presenting test line combinations of RDTs with interpretations listed in a multiple choice format and (ii) a questionnaire about their practices of malaria diagnosis.

**Results:**

Among 135 subscribing laboratories, 134 (99.3%) used 139 RDT products (11 different products from 8 brands). After exclusion of the results of one laboratory, analysis was done for 133 laboratories using 137 RDT products. Scores of 10/10, 9/10 and 8/10 were achieved for 58.4%, 13.1% and 8.0% of 137 RDT products respectively. For three-band *P*. *falciparum*–pan-*Plasmodium* RDTs (113 (82.5%) products, 6 brands), most frequent errors were (1) disregarding faint test lines (18.6%), (2) reporting invalid instead of *P*. *falciparum* (16.8%) and (3) reporting “*Plasmodium* spp., no further differentiation possible” without mentioning the presence or absence of *P*. *falciparum* (11.5%). For four-band RDTs (21 (15.3%) products, 1 brand), errors were (4) disregarding faint *P*. *vivax* test lines (47.6%) and (5) reporting “*Plasmodium* spp., no further differentiation possible” without mentioning the presence of *P*. *falciparum* and *P*. *vivax* (28.6%). Instructions for use (IFU) of only 4 out of 10 RDT products mentioned to interpret faint-intensity test lines as positive (conducive to errors 1 and 4) and IFU of 2 products displayed incorrect information (conducive to errors 2 and 5). Outside of office hours, 36.1% of participants relied on RDTs as the initial diagnostic test; 13.9% did not perform microscopic confirmation.

**Conclusion:**

Reading and interpretation of malaria RDTs was satisfactory, but errors were embedded in the instructions for use of the products. Relying on RDTs alone for malaria diagnosis (about one third of participants) is not a recommended practice.

## Introduction

Malaria is a disease caused by the parasite *Plasmodium*, which is transmitted by the bites of the female infected *Anopheles* mosquito. There are five *Plasmodium* species affecting humans: *Plasmodium falciparum* (occurring in tropical regions and representing the predominant species in Africa), *Plasmodium vivax*, *Plasmodium ovale*, *Plasmodium malariae* and *Plasmodium knowlesi* [1, 2, 3]. In 2015, malaria caused 429 000 deaths worldwide, mostly caused by *P*. *falciparum* (99%) in children aged under 5 years (70%) [4]. The prevalence of malaria in Belgium was 3.2 and 3.3 per 100 000 inhabitants in 2015 and 2016, respectively.

Imported malaria is a rare but potentially fatal event: in the UK, 1300 to 1800 malaria cases are yearly reported with 2–11 deaths [1]. In non-immune travellers, *P*. *falciparum* malaria can quickly evolve to life-threatening complications. Outcome depends on timely diagnosis and prompt treatment [1,5,6,7,8,9]. As symptoms of malaria are non-specific, diagnosis depends on the laboratory: Giemsa-stained thin and thick blood films provide–in addition to the diagnosis of malaria—also information about the involved *Plasmodium* species and parasite density. This information is relevant for reasons of selection of antimalarial drugs and hospital admission (*P*. *falciparum*) and eradication of liver forms (*P*. *vivax* and *P*. *ovale*) [1]. In addition, parasite densities (expressed as number of asexual parasites/μl of blood or as number of infected red blood cells/μl of blood or as a percentage of total red blood cells) are a laboratory indicator for severity and are used for follow-up of treatment [1].

Malaria microscopy requires considerable expertise [10, 11]. In non-endemic settings, this expertise is increasingly rare due to low exposure to positive samples, particularly outside of office hours [1, 10]. In these situations, malaria rapid diagnostic tests (RDTs) present a valuable adjunct to microscopy [1,10, 12, 13]. RDTs are simple, hand-held diagnostic devices (mostly cassettes) that detect *Plasmodium* antigens in the blood. The present antigens migrate along a nitrocellulose strip and are captured by *Plasmodium*-specific antibodies coupled to a signal (mostly colloidal gold) which generates a visible cherry-red test line that appears within 30 minutes. RDTs also provide a so-called control line which indicates correct migration. Depending on the number of lines (“bands”) that appear on the strip in the cassette (the control line plus respectively one, two or three “test lines” on which signals appear), RDTs are categorized as “two-, three-, or four-band RDTs, see Table 1) [13,14].

**Table 1:** Rapid diagnostic test products used by the participants (137 RDT products from 8 brands, used by 133 participants). The underlined names represent the shortened names used in the text to refer to the different RDT products.

Manufacturer Product Antigens detected	Product passed performance criteria of WHO round testing? (latest WHO malaria RDT round in which product was tested)	Number of RDTs used (%)
Three-band tests Pf–pan (Pf, Pv, Pm, Po)		Total = 113 (82.5)
Alere Scarborough Inc, Scarborough, Maine, USA Binax Now® Malaria Test Pf-HRP2, (pan-)aldolase	No (Round 1) (Delisted from summary rounds 1–7. Not eligible for WHO procurement—2018)	59 (43.1)
Standard Diagnostics Inc, Hagal-Dong, Korea SD BIOLINE Malaria Ag P.f./Pan (05FK60) Pf-HRP2, pan-pLDH	Yes (Round 5)	18 (13.1)
Access Bio Inc, New Jersey, USA CareStart^TM^ Malaria HRP2/ pLDH Combo Test Pf-HRP2, pan-pLDH	Yes (Round 5)	14 (10.2)
nal von minden GmbH, Regensburg, Germany NADAL® Malaria Test Pf-HRP2, pan-pLDH	Product not included in WHO round testing	3 (2.2)
Cypress Diagnostics, Leuven, Belgium Malaria Total Quick Test Pf-HRP2, pan-pLDH	Product not included in WHO round testing	2 (1.5)
Bio-Rad, Marnes-la-Coquette, France OptiMAL-IT Pf-pLDH, pan-pLDH	No (Round 3) (Delisted from summary rounds 1–7. Not eligible for WHO procurement– 2018)	14 (10.2)
Access Bio Inc, New Jersey, USA CareStart^TM^ Malaria pLDH 3 Line Test Pf-pLDH, pan-pLDH	No (Round 7) (Delisted from summary rounds 1–7. Not eligible for WHO procurement– 2018)	2 (1.5)
Standard Diagnostics Inc, Hagal-Dong, Korea SD BIOLINE Malaria Ag (05FK40) Pf-pLDH, pan-pLDH	No (Round 3) (Delisted from summary rounds 1–7. Not eligible for WHO procurement—2018)	1 (0.7)
Four-band tests Pf, Pv, pan (Pf, Pv, Pm, Po)		Total = 21 (15.3)
All Diag, Strasbourg, France PALUTOP+4 OPTIMA® Pf-HRP2, Pv-pLDH, pan-pLDH	Yes (Round 7)	21 (15.3)
Two-band tests Pf or Pv		Total = 3 (2.2)
ulti med Products GmbH, Ahrensburg, Germany Malaria Plasmodium falciparum Test Pf-HRP2	Product not included in WHO round testing	1 (0.7)
Standard Diagnostics Inc, Hagal-Dong, Korea SD BIOLINE Malaria Ag P.v (05FK70) Pv-pLDH	Yes (Round 2) (Delisted from summary rounds 1–7. Not eligible for WHO procurement—2018)	2 (1.5)
TOTAL		137 (100)

Pf = *Plasmodium falciparum*, Pv = *Plasmodium vivax*, Pm = *Plasmodium malariae*, Po = *Plasmodium ovale*, pan = all *Plasmodium* species (Pf, Pv, Pm, Po), HRP 2 = histidine-rich protein II, pLDH = *Plasmodium* lactate dehydrogenase.

External Quality Assessment (EQA) of malaria RDTs remains difficult to perform on clinical blood samples as high volumes of blood are needed to be distributed to a large number of participants [15]. The present study reports the results of a photograph-based EQA organised among diagnostic laboratories in a non-endemic country and focuses on (i) reading and interpretation of RDTs as well on their (ii) actual use in the diagnostic algorithm.

## Materials and methods

### Ethics statement

The rapid diagnostic tests used to create the photographs for this study were obtained from patient’s tests. The ITM travel clinic adopted the presumed consent or opt-out procedure by which a written objection is given if they do not wish their biospecimens to be retained for research (or data used for research). The study protocol had been approved by the ITM institutional review board on 4^th^ June 2013.

Concerning the information of the patients, this work was conducted following the ISO 17043 standard indicating that: “the identity of participants was known only to people involved in coordinating the EQA programme, and was maintained confidential”. The Institute of Public Health is accredited ISO17043.

### Participants

Participants were diagnostic laboratories in Belgium and the Grand Duchy of Luxembourg which participated to the EQA (Proficiency Testing) program of microscopic malaria examination previously organised by the Institute of Public Health (IPH), Brussels, Belgium. Prior to the survey, diagnostic laboratories (further referred to as “participants”) were asked which malaria RDTs product(s) they used; a total of 11 different RDT products were used. These are all commercial tests.

1. CareStart Malaria HRP2/pLDH (pf/pan) Combo test (AccessBio, Carlsbad, California, USA)
2. Carestart pLDH 3 line malaria (accessBio, Carlsbad, California, USA)
3. Binax Now malaria (Alere Health, Scarborough, Maine, USA)
4. Palutop 4+ optima (All Diag, biosynex, Illkirch-Graffenstaden, France)
5. OptiMAL-IT (BioRad, Hercules, California, USA)
6. Malaria Total (P.f., P.v., P.m., P. o.) (Cypress diagnostics, Langdorp, Belgium)
7. Nadal Malaria Test (Nal von Minden, Regensburg, Germany)
8. SD Bioline Malaria Ag P.f./Pan (FK60) (Standard Diagnostics, Alere, Scarborough, Maine, USA)
9. SD Bioline Malaria Ag P.v. (FK70) (Standard Diagnostics, Alere, Scarborough, Maine, USA)
10. SD Bioline Malaria Ag P.f./Pan (FK40) (Standard Diagnostics, California, USA)
11. Malaria (plasm. Falciparum)-test cassette (Ulti Med, Ahrensburg, Germany)

### Photograph-based samples of malaria rapid diagnostic tests

**Fig 1:**
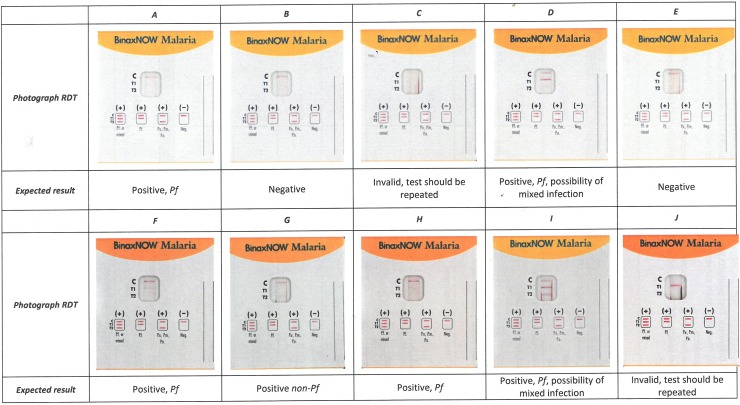
Example of photographs for Alere Binax Now® Malaria Test, (n = 59). Abbreviations: Pf = *P*. *falciparum*, Pm = *P*. *= malariae*, Po = *P*. *ovale*, non-Pf = *Plasmodium* non-*falciparum*, IFU: Instructions for use.

The EQA consisted of high-resolution photographs (macro silk normal full colour CMYK, 300dpi, Bulckens, Herenthout, Belgium) in real-life dimensions presenting combinations of control and test lines at different line intensities. The reference (correct) interpretation was embedded in a multiple choice list of six options. Separate sets of photographs were made for each of the 11 RDT products and each laboratory received the set of photographs corresponding to the RDT product(s) it used. Two sets of photographs were used for multiple RDT products (one for three RDT products and one for two RDT products). Fig 1 shows the set of photographs for one of the RDT products.

### Questionnaire

In addition to the set of photographs, participants received a questionnaire about their practice of malaria diagnosis in particular with regard to the place of RDTs in the diagnostic algorithm for malaria.

### EQA validation and session

Prior to distribution, photographs, questions and questionnaire were validated by an expert panel of participants, in line with IPH EQA procedures. The IPH sent out the EQA on September 3^rd^ 2013 and participants were asked to return their (paper-based) results by post mail before September 17^th^ 2013. Results were subsequently entered in an Excel spread sheet (Microsoft Corporation, Redmond, Washington, USA).

### Scoring of the answers to the photographs

Answers submitted by the participants were compared to the reference results. Errors were categorised as major or minor depending on their potential impact on patient care. Major errors included (i) missing the diagnosis of malaria, (ii) not reporting the presence of *P*. *falciparum*, or (iii) missing an invalid result. Minor errors included (i) not reporting the presence of *Plasmodium* non-*falciparum* (*i*.*e*. the absence of *P*. *falciparum*) when reporting malaria, (ii) not reporting the possibility of a mixed *Plasmodium* species infection, (iii) reporting an incorrect species identification for non-*falciparum* species or (iv) reporting a negative result as positive.

## Results

### Participation rate, overview of participants and products used

Out of the 172 diagnostic laboratories which performed malaria diagnosis in 2013, 135 (78.5%) had declared to use a total of 11 different RDTs and were subscribed to the RDT EQA; participation rate was 99.3% (134/135). Most participants (130/134, 97.0%) used one RDT brand; three laboratories used two RDT products of different brands and one laboratory used three RDT brands. One laboratory reported combined answers for the two RDT products used–this answer was subtracted from analysis. As a result, the total number (denominator) of interpretable results for the photographs was 137 (numbers of RDT products used) from 133 participants and for the survey 132 (numbers of laboratories, response rate 97.8%); Table 1 gives an overview of the different RDT products used. Most used products (113/137, 82.5%) were three-band RDTs, consisting of a control line, a *P*. *falciparum* line and a pan-*Plasmodium* line (detecting antigens common to all *Plasmodium* species). The four-band RDT (one product) represented 21 (15.3%) of RDTs used; this RDT detects antigens specific to *P*. *falciparum* and *P*. *vivax* as well as a pan-*Plasmodium* antigen. Other RDT products were rare and included a two-band *P*. *vivax* RDT (2 participants, product used in addition to other RDT products) and a single two-band *P*. *falciparum* RDT.

### Overall scores

Overall (n = 137), results were good to very good. Scores of 10/10, 9/10 and 8/10 were achieved for 58.4% (n = 80), 13.1% (n = 18) and 8.0% (n = 11) of 137 RDT products respectively; the lowest score was 5 (1 participant). There were no errors (major nor minor) for the SD BIOLINE Malaria Ag P.v (05FK70) or the SD BIOLINE Malaria Ag (05FK40).

### Scores for the three-band RDTs (113 RDT products from 6 brands)

For the main type of RDT used, *i*.*e*. the three-band *P*. *falciparum*–pan-*Plasmodium* detecting RDT, two-thirds of RDT products (75/113, 66.4%) obtained the maximum score. The most frequently reported major error was disregarding a faint *P*. *falciparum* test line and reporting a negative result (21/113, 18.6% of RDT products). This error was made by 2/16 CareStart^TM^ HRP2/pLDH Combo Test users, 1/3 NADAL® Malaria Test users and 10/14 OptiMAL users but by none of the participants using Binax Now®. Although the *P*. *falciparum* test lines of the concerned products appeared slightly weaker compared to the Binax Now® product, they were still visible. Reviewing the instructions for use (IFU) showed that IFUs of only 4 out of 11 RDT products mentioned to consider test lines of weak or faint intensity as positive (Binax Now®, PALUTOP, Malaria Total Quick Test and ulti med).

**Fig 2:**
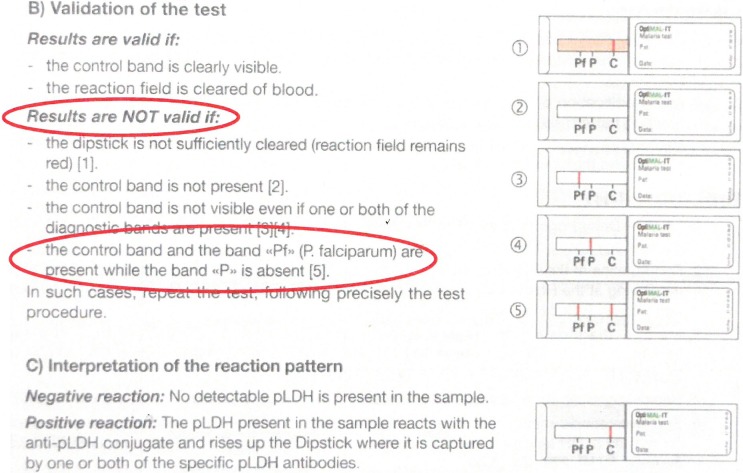
Instructions for Use (IFU) for Bio-Rad OptiMAL-IT, (881056, version 01/2010, included in each box containing 24 individual test packs). The IFU did not clearly mention the (rare) possibility of a *P*. *falciparum* infection with only a Pf-pLDH band without a pan-pLDH band. According to the IFU, the test result should be reported as invalid.

The second major error was missing the diagnosis of malaria by reporting invalid instead of *P*. *falciparum* malaria (19/113, 16.8%). This error occurred among all but one (13/14) OptiMAL users. Of note, the OptiMAL IFU mentioned that in case of co-presence of the control line and *P*. *falciparum*- (Pf-pLDH) test line without a visible pan-*Plasmodium* (pan-pLDH) test line, the result should be interpreted as “invalid” (Fig 2).

In addition, “*Plasmodium* spp., no further differentiation possible” was answered 13 times (13/113, 11.5%), however this answer did not disclose the presence (both *P*. *falciparum* and pan-*Plasmodium* test lines visible, n = 2) nor absence (no *P*. *falciparum* but pan-*Plasmodium* test line visible, n = 11) of *P*. *falciparum* (representing major and minor errors respectively. Finally, there were 16 minor errors (16/113, 14.2%) related to the interpretation of the presence of both *P*. *falciparum* and non-*falciparum* test lines, for which the correct answer is “*P*. *falciparum*, possibility of a mixed infection”: errors occurred in both senses (*i*.*e*. not mentioning the possibility of mixed infection when both lines were present (n = 6) as well as mentioning the possibility of mixed infection in the case of only the *P*. *falciparum* line visible (n = 10)). Additional errors occurred sporadically among products and participants and were random, suggesting clerical errors.

### Scores for the four- and two- band RDTs (21 RDT products from 1 brand and 3 RDT products from 2 brands respectively)

**Fig 3:**
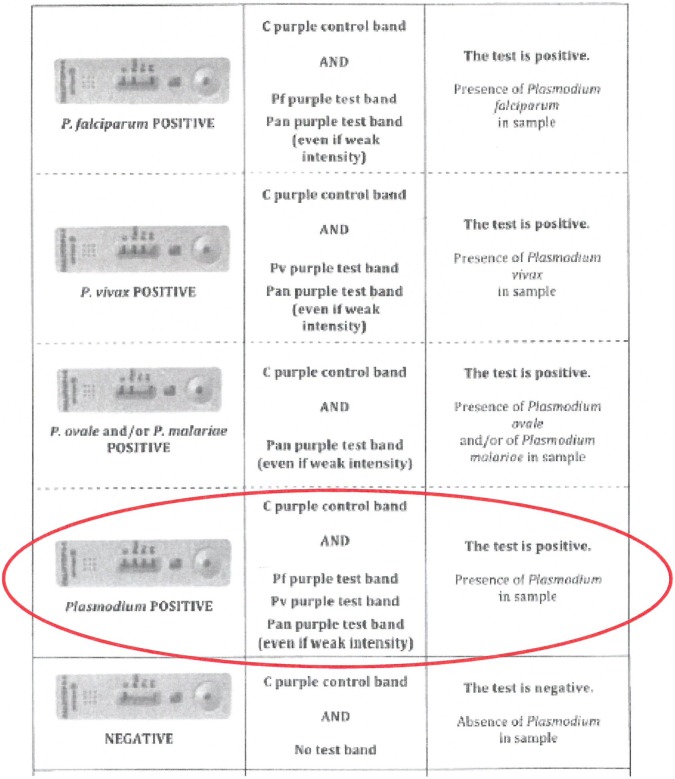
Instruction for Use (IFU) for All Diag PALUTOP+4 OPTIMA® (version 10, 03/07/2012). The possibility of a mixed infection of *P*. *falciparum* and *P*. *vivax* with *P*. *ovale* and/or *P*. *malariae* was not clearly mentioned in the IFU (not included in “8.1 Test results”, but further under “Test limits”).

Among the participants using the four-band PALUTOP, nearly half (47.6%) missed a faint test line and reported negative instead of *P*. *vivax*–which represented a major error. Another major error (committed by more than a quarter (28.6%) of participants) consisted of reporting “*Plasmodium* spp., no further differentiation possible” instead of mentioning the presence of both *P*. *falciparum* and *P*. *vivax*. The latter error was embedded in the IFU, which mentioned to report “Presence of *Plasmodium*” for this combination of test lines (Fig 3). There were four additional major errors (apparently at random) as well as 31 minor errors, most of which were related to not fully exploiting the pattern of combined test lines indicating for instance the presence or absence of *P*. *vivax* or *P*. *falciparum* mixed infection. In retrospect, part of these errors could have been suggested by the options for answering, another part was embedded in the IFU (Fig 3).

For the two-band ulti med product, the single user reported two negative results instead of *P*. *falciparum* (major error, Photo A as well G, faint test line). The two users of the SD BIOLINE Malaria Ag P.v (05FK70) test did not make any errors.

In total there were 28 minor and 59 major errors for the 3-band tests; 32 minor and 21 major errors for the 4-band tests.

### Results of the questionnaire: Practice of malaria diagnosis

The number of requests for malaria diagnosis as well as the exposure per laboratory technician was low, with 112 (84.8%) of 132 participants processing ≤ 100 and 46 (34.8%) even ≤ 20 requests per year. Most participants (92.4%, 122/132) declared to perform malaria diagnosis during as well as outside of office hours, but the strategy differed: during office hours, most (97.0%, 128/132) performed microscopy in combination with a RDT (always or as a confirmatory test in case of microscopic detection of malaria parasites by 84.8% (122/132) and 12.1% (16/132) of participants respectively). A minority of participants left the decision about performing RDT versus microscopy to the clinician or used microscopy only for confirmation (1.5% (2/132) of participants each). Outside of office hours, 85.2% (104/122) of participants consistently performed microscopy (always or as a confirmatory test for 65.6% (80/122) and 19.7% (24/122) of participants respectively). More than one third (44/122, 36.1%) of participants relied on the RDT as an initial test; 13.9% (17/122) used the RDT as the only diagnostic tool and 22.1% (27/122) provided microscopy for confirmation (of which six always and 21 only in case of doubt).

## Discussion

The present EQA session assessed the competence of reading and interpretation of malaria RDTs in a non-endemic setting and surveyed their use in the diagnostic strategy. More than three-quarters (78.5%) of laboratories performing diagnosis of malaria declared to use malaria RDTs, which is slightly more compared to a previous survey in the same area (72.7%) [15]. These proportions are higher than previously noted in the UK (44.3%) and the US (17%), [5, 16]. Participants confirmed the low number of requests for malaria diagnosis, in line with findings elsewhere in non-endemic settings [16, 17, 18]. Following WHO, “current evidence indicates that use of microscopy and RDTs is sufficient for clinical management of patients with suspected malaria, routine surveillance and passive case detection in low-transmission areas. NAA-based diagnostic methods are not required for these applications”. *P*. *falciparum*/non-*falciparum* three-band products were the most widely used products (more than three-quarters of participants).

Overall results of reading and reporting were good to very good. A major error–occurring among 18.6% and 47.6% of three- and four-band product users respectively—was disregarding the presence of faint test lines and reporting the result as negative. Although this incorrect answer was noted mostly for RDT products for which photos displayed the faintest lines, the present EQA design did not allow to trace whether this error was related to observation versus interpretation. Faint test lines of malaria RDTs should be interpreted as positive [19, 20]. Weak and faint test line intensities can be expected at low parasite densities (at which non-immune persons such as travelers may develop disease [13, 17, 21] and exceptionally–in the case of the *P*. *falciparum* HRP2 antigen, as part of a prozone effect [22, 23, 24]. In endemic settings, disregarding faint test lines is a commonly observed user error [19, 25,26]. Given the potential impact of disregarding faint test lines and reporting them as negative, it is striking that IFUs of less than half of the RDTs alerted the user about reading and interpretation of faint test lines.

Two other major errors were associated with incorrect IFU information about the interpretation of combinations of test lines *i*.*e*. reporting an invalid result in case of a *P*. *falciparum* (OptiMAL) and reporting “Presence of *Plasmodium”* without mentioning the presence of *P*. *falciparum* and *P*. *vivax* in the case of PALUTOP. This is striking, as for both RDT products, errors in the IFU had been noted previously [15]. These observations are of concern: the IFU of in-vitro diagnostics must clearly point to all residual risks (*i*.*e*. risks that cannot be mitigated by adaptations and improvement of product design) of product failure and test limitations [27, 28].

Another observation of this EQA was that the information about the presence (or absence) of *P*. *falciparum* was not fully exploited, for instance by reporting “*Plasmodium spp*., no further differentiation possible” instead of either *P*. *falciparum* or *Plasmodium* non-*falciparum* (11.6% among the three-band users). Indeed, an asset of RDTs is their ability to rule in or out the presence of *P*. *falciparum* and this conveys important information to the clinician. Part of these errors may have been caused by non-familiarity with the options for answering the EQA questions; alternatively operator misunderstanding or misinterpretation of test line patterns is a known limitation of RDTs also in non-endemic settings [10].

The survey further revealed that, outside of office hours, more than one third (36.1%) of participants relied on the RDT either as the single diagnostic tool (13.9%) or with subsequent microscopy (22.1%). This observation is in line with what was previously recorded in the same setting [15]. Given the non-perfect performance of RDTs (and in particular the false-negatives at low *P*. *falciparum* densities), most authors recommend to use RDTs as a complement and not as substitute for microscopy in non-endemic settings [1, 10,17,25,29, 30]. Alternatively, if used as an initial diagnostic tool, recommendations clearly state to confirm all negative RDT results by microscopy as soon as possible and–pending this confirmation—to keep the patient under medical surveillance including assessment of any clinical danger sign or critical laboratory indicator such as thrombocytopenia [12,31].

Our study had some limitations. First, the photograph-based design did not allow to assess the pre-analytical and large parts of the analytical phase (including the operator’s and RDT’s performance). Second, as stated above, the EQA design did not allow to trace errors to either misreading of test lines versus misinterpretation. Further, the idea of offering each participant with “their own” RDT product conveyed the challenge of harmonizing panels and test line intensities among the different RDT products. Next, non-familiarity with pre-formatted answers may explain some anecdotal errors which in retrospect might have been clerical (*e*.*g*. reporting *P*. *falciparum* in the case of a clearly negative test result). Among the strengths, there was the high response rate (99.3%) in the present EQA and questionnaire versus survey response rates of 60.3% and 72.3% in the United Kingdom and the US respectively [5,16]. Likewise, by offering a panel of standardized photographs, the present EQA allowed to focus on the reading and interpretation—which was identified as a possible source of error during a previous EQA [15] and to trace errors to product IFUs.

Although not intended nor designed as a study objective, it is tempting to compare the present EQA results with those obtained previously in a low-resource setting (Democratic Republic of the Congo (DRC) [14,32]. Although occurring in lower proportions in the present setting, there were striking similarities such as recognition of invalid or negative results, difficulty in reading and/or interpretation of faint test lines and species differentiation. Apart from other reasons (training, availability of and compliance to standard operating procedures, etc.), differences in proportions may be explained by the fact that the EQAs in DRC addressed individual healthcare workers and not the diagnostic laboratory as an entity.

In conclusion, the present EQA results showed that diagnostic laboratories performed well in reading and interpretation of malaria RDTs. Systematic errors comprised disregarding faint test lines and reporting them as negative as well as interpretation errors of some test line patterns, part of these errors were embedded in the instructions for use of the products. Further, the information about presence or absence of *P*. *falciparum* (one of the assets of RDTs) was not always exploited. Relying on RDTs alone for malaria diagnosis was practiced by nearly one third of participants but is not a recommended practice.

## Supporting information

S1 Fig Original questionnaire. (PDF)

S1 Text English translation of the original questionnaire. (DOCX)
