# Supplementary material for: Photo-based External Quality Assessment of Malaria rapid diagnostic tests in a non-endemic setting
Source: PLoS One. 2018 Aug 31;13(8):e0201622. doi: 10.1371/journal.pone.0201622 (PMC6118386; doi:10.1371/journal.pone.0201622)
Supplement: S1 Fig — (PDF) [file pone.0201622.s001.pdf]

EXTERNE KWALITEITSEVALUATIE: PARASITOLOGIE
Rapid Diagnostic Tests (RDT) voor malaria
Vragenlijst

Cyclus :

2013/1

Datum van ontvangst :

Resultaten binnen te sturen voor :

17/09/2013

WIV, dienst Kwaliteit van medische laboratoria
J. Wytsmanstraat, 14
1050 Brussel
Tel. : 02/642.55.22 Fax. : 02/642.56.45

Vul voor uw laboratorium de volgende vragen in:

1. In het voorbije jaar (2012) hadden wij voor Malaria diagnostiek een aantal aanvragen tussen :

- ☐ 0 - 10
- ☐ 11 - 20
- ☐ 21 - 100
- ☐ 101 - 500
- ☐ meer dan 500

2. Wij voeren malaria diagnostiek uit :

- ☐ Tijdens de « kantooruren » (reguliere openingsuren van het laboratorium)
- ☐ Buiten de « kantooruren » (avond, nacht en/of weekend)
- ☐ Zowel tijdens als buiten de « kantooruren »

3. Malaria diagnostiek in ons laboratorium TIJDENS de kantooruren bestaat uit :

- ☐ Uitsluitend microscopie
- ☐ Uitsluitend malaria sneltest
- ☐ Microscopie EN malaria sneltest in ALLE gevallen
- ☐ Steeds microscopie, bij twijfel of voor bevestiging : malaria sneltest
- ☐ Steeds malaria sneltest, bij twijfel of indien positief : microscopie
- ☐ Malaria sneltest indien aangevraagd door clinicus
- ☐ Microscopie indien aangevraagd door clinicus
- ☐ Andere : ....
....

4. Malaria diagnostiek in ons laboratorium BUITEN de kantooruren bestaat uit :

- ☐ Uitsluitend microscopie
- ☐ Uitsluitend malaria sneltest
- ☐ Microscopie EN malaria sneltest in ALLE gevallen
- ☐ Steeds microscopie, bij twijfel of voor bevestiging : malaria sneltest
- ☐ Steeds malaria sneltest, bij twijfel of indien positief : microscopie
- ☐ Malaria sneltest indien aangevraagd door clinicus
- ☐ Microscopie indien aangevraagd door clinicus
- ☐ Andere : ....
  ....

5. Het aantal laboratorium technologen dat malaria sneltests uitvoert (TIJDENS en BUITEN de kantooruren) ligt tussen :

- ☐ 0 - 5
- ☐ 6 - 10
- ☐ 11 - 15
- ☐ 16 - 20
- ☐ meer dan 20

6. Het aantal malaria sneltesten dat wij vorig jaar (2012) uitvoerden ligt tussen :

- ☐ 0 - 10
- ☐ 10 - 20
- ☐ 21 - 100
- ☐ 101 - 500
- ☐ meer dan 500

7. Wij gebruiken volgende test voor malariasneltesten :

☐ (zie lijst met codes in bijlage)

8. Wij gebruiken deze test sinds :

- ☐ Minder dan 1 jaar
- ☐ Tussen 1 en 3 jaar
- ☐ Meer dan 3 jaar
- ☐ Meer dan 5 jaar

9. Om de sneltest uit te voeren brengen wij het benodigde volume bloed aan :

- ☐ Door gebruik te maken van het systeem dat zich in de kit bevindt (plastieken pipet, loop, inverted cup)
- ☐ Door gebruik te maken van een automatische pipet

10. Hebt u in de periode tussen 2010 en 2012 uw methode van sneltesten gewijzigd :

- ☐ Ja
- ☐ Neen

10.1 Indien ja, welk test gebruikte u voordien : ☐ (zie lijst met codes in bijlage)

10.2 Waarom bent u van methode veranderd :

....

....

....

....

11. Wij stuurden vorig jaar (2012) de volgende stalen naar het referentielaboratorium (meer dan 1 antwoord kan aangevinkt worden) :

- ☐ Geen
- ☐ Stalen waarover twijfel i.v.m. de diagnose was
- ☐ Stalen met microscopische diagnose van malaria
- ☐ Stalen met positieve antigen test
- ☐ Stalen met een discordant resultaat tussen de microscopie en de antigen test

Datum : ....

Naam en handtekening : ....

EVALUATION EXTERNE DE LA QUALITE: PARASITOLOGIE
Test de Diagnostic Rapide (TDR) du paludisme
Questionnaire

Cycle :

2013/1

Date de réception :

Résultats à renvoyer avant le :

17/09/2013

ISP, service Qualité des laboratoires médicaux
Rue J. Wytsman, 14
1050 Bruxelles
Tél. : 02/642.55.21 Fax. : 02/642.56.45

Répondez aux questions suivantes pour votre laboratoire :

1. Durant l'année écoulée (2012), le nombre de demandes que nous avons reçues pour diagnostic du paludisme était compris entre :

- ☐ 0 - 10
- ☐ 11 - 20
- ☐ 21 - 100
- ☐ 101 - 500
- ☐ Plus de 500

2. Nous réalisons le diagnostic du paludisme :

- ☐ Durant les « heures de bureau » (heures d'ouverture régulière du laboratoire)
- ☐ En dehors des « heures de bureau » (soirée, nuit et/ou weekend)
- ☐ Aussi bien durant qu'en dehors des « heures de bureau »

3.DURANT LES HEURES DE BUREAU, nous effectuons le diagnostic du paludisme sur base :

- ☐ De la microscopie uniquement
- ☐ D'un test rapide paludisme uniquement
- ☐ De la microscopie ET d'un test de diagnostic rapide paludisme, dans TOUS les cas
- ☐ De la microscopie, dans TOUS les cas suivi d'une confirmation en cas de doutes par un test de diagnostic rapide
- ☐ D'un test de diagnostic rapide dans TOUS les cas, suivi en cas de doutes ou de résultat positif par un examen microscopique
- ☐ D'un test de diagnostic rapide malaria si demandé par le médecin
- ☐ De la microscopie si demandée par le médecin
- ☐ Autre : ....
....

4. EN DEHORS DES HEURES DE BUREAU, nous effectuons le diagnostic du paludisme sur base :

- ☐ De la microscopie uniquement
- ☐ D'un test rapide paludisme uniquement
- ☐ De la microscopie ET d'un test de diagnostic rapide paludisme, dans TOUS les cas
- ☐ De la microscopie, dans TOUS les cas suivi d'une confirmation en cas de doutes par un test de diagnostic rapide
- ☐ D'un test de diagnostic rapide dans TOUS les cas, suivi en cas de doutes ou de résultat positif par un examen microscopique
- ☐ D'un test de diagnostic rapide malaria si demandé par le médecin
- ☐ De la microscopie si demandée par le médecin
- ☐ Autre : ....
....

5.Le nombre de techniciens de laboratoire qui réalisent des tests de diagnostic rapide du paludisme PENDANT ET DEHORS DES HEURES DE BUREAU est compris entre :

- ☐ 0 - 5
- ☐ 6 - 10
- ☐ 11 - 15
- ☐ 16 - 20
- ☐ Plus de 20

6. Le nombre de tests de diagnostic rapide paludisme que nous avons réalisés l'année passée (2012) est compris entre :

- ☐ 0 - 10
- ☐ 10 - 20
- ☐ 21 - 100
- ☐ 101 - 500
- ☐ Plus de 500

7. Nous utilisons le test de diagnostic rapide suivant :

☐ (voir liste avec les codes en annexe)

8. Nous utilisons les tests de diagnostic rapide depuis :

- ☐ Moins d' 1 an
- ☐ Entre 1 et 3 ans
- ☐ Plus de 3 ans
- ☐ Plus de 5 ans

9. Pour réaliser le test rapide, nous transférons le volume nécessaire de sang :

- ☐ En utilisant le système inclus dans le kit (pipette en plastique, loop, inverted cup)
- ☐ En utilisant une pipette automatique

10. Entre les années 2010 et 2012, avez-vous changé de test de diagnostic rapide du paludisme ?

- ☐ Oui
- ☐ Non

10.1 Si oui, quel était l'ancien test utilisé : ☐ (voir liste avec les codes en annexe)

10.2 Pourquoi avez-vous changé de format de test de diagnostic rapide du paludisme ?

....

....

....

....

11. Nous avons envoyé l'année passée (2012) au laboratoire de référence malaria (plusieurs réponses possible) :

- ☐ Aucun échantillon
- ☐ Les échantillons qui posaient des problèmes de diagnostic
- ☐ Les échantillons microscopiquement positifs pour le paludisme
- ☐ Les échantillons avec un test rapide postif
- ☐ Les échantillons avec un resultat discordant entre la microscopie et le test de diagnostic rapide

Date : ....

Nom et signature : ....
