# Supplementary material for: Photo-based External Quality Assessment of Malaria rapid diagnostic tests in a non-endemic setting
Source: PLoS One. 2018 Aug 31;13(8):e0201622. doi: 10.1371/journal.pone.0201622 (PMC6118386; doi:10.1371/journal.pone.0201622)
Supplement: S1 Text — (DOCX) [file pone.0201622.s002.docx]

External Quality Assessement: Parasitology

Rapid Diagnosic Tests (RDT) Paludism

Please answers de following questions for your laboratory.

1. During 2012, how many demands have you received for the diagnosis of paludism?
2. We performed the paludism diagnosis

- During the normal office hours
- Outside the normal office hours
- Both

1. During the office hours we performed the paludism diagnosis based on:

Microscopy only

RDT only

Microscopy and RDT in all cases

Microscopy in all cases followed by a confirmation with RTD in case of doubt

RDT in all cases followed by microscopy in case of doubt or positive result

RDT malaria if requested by the clinician

Microscopy if requested by the clinician

Other

1. Outside the of office hours we performed paludism diagnosis based on (idem 3)
2. The number of technicians performing the RDT malaria within and outside the office hours
3. The number of RDT malaria performed during last year (2012)
4. We used the following test (see list in annex)
5. We used RDT since
   1. Less than 1 year
   2. Between 1 and 3 years
   3. More than 3 years
   4. More than 5 years
6. To perform the RDT, we transfer the required blood volume using:
   1. The system provided with the kit
   2. Using a micropipette
7. Between 2010 and 2012, did you change your RDT malaria?
8. We have sent last year to the reference laboratory (several answers possible):

No sample

Sample with diagnostic problems

Samples positive in microscopy

Sample positive with RDT

Samples with discordant results between microscopy and RDT

-
